# Reliability of the CPAK classification assessed on long-leg X-rays in patients undergoing total knee arthroplasty

**DOI:** 10.1186/s42836-026-00381-y

**Published:** 2026-04-02

**Authors:** Xavier Gasparutto, Daniel Chantre, Alice Bonnefoy-Mazure, Pierre-Alban Bouché, Didier Hannouche, Stéphane Armand, Hermès Howard Miozzari

**Affiliations:** 1 Kinesiology Laboratory, Geneva University Hospitals and University of Geneva, 1205 Geneva, Switzerland; 2 Division of Orthopedic Surgery and Musculoskeletal Trauma Care, Geneva University Hospitals, 1205 Geneva, Switzerland; 3 Centre of Research On Skeletal Muscle and Movement, Geneva University Hospitals and University of Geneva, 1205 Geneva, Switzerland

**Keywords:** Arthroplasty, Replacement, Knee, Alignment, CPAK, Phenotype

## Abstract

**Background:**

Patient-specific knee alignment is regarded as a major target for improving total knee arthroplasty (TKA) outcomes. The Coronal Plane Alignment of the Knee (CPAK) classification has been proposed to capture the native patient-specific knee alignment, according to the Joint Line Obliquity (JLO) and arithmetic Hip Knee Ankle angle (aHKA), themselves based on the Lateral Distal Femoral Angle (LDFA) and Medial Proximal Tibial Angle (MPTA). This study aims to evaluate intra-operator, inter-operator, and test–retest reliability of the CPAK classification and associated angles in both knees with osteoarthritis (KOA) and TKA.

**Methods:**

From our local arthroplasty registry, patients who sequentially underwent TKA on both knees within 18 months, with long-leg X-rays before and after each surgery between 2018 and 2023, were retrospectively selected. The contralateral knees before the 1st and 2nd TKA were used as test–retest for KOA and TKA knee, respectively. Four operators with increasing experience performed two measures of MPTA and LDFA for each image. The intra-operator, inter-operator, and test–retest reliability were assessed with Intraclass Correlation Coefficient (ICC(3,1)), Smallest Detectable Change (SDC), and Cohen’s Kappa.

**Results:**

The study included 34 patients. Angles showed good to excellent ICC apart from JLO in the KOA condition (moderate). Measures of LDFA on KOA and all TKA angles presented good to excellent SDC (< 3°), including test–retest conditions. MPTA, JLO, and aHKA on KOA showed moderate SDC (< 4.2°). CPAK classification was moderate to substantial for KOA (Kappa of 0.5 to 0.64) and substantial to almost perfect for TKA (Kappa of 0.69 to 0.81). Reliability increased with experience.

**Conclusions:**

For experienced operators, CPAK classification and associated angles demonstrated levels of inter-rater reliability acceptable for clinical use in knees with TKA but at the limit of acceptability for knees with severe OA. In severe OA, one should interpret CPAK types cautiously, and angles may be preferable. MPTA in the knees with OA appeared as the main factor undermining reliability. Clarification on this angle may be needed to improve reliability, especially when using philosophies aiming at restoring the native alignment. Finally, test–retest reliability levels suggested that these measures are appropriate for longitudinal assessment.

## Introduction

Patient-specific knee alignment is regarded as a key step for improving total knee arthroplasty (TKA) outcomes in the future [1–3], due to the variability of healthy and osteoarthritic knee anatomy [4, 5]. Besides the mechanical alignment, various alignment techniques are now considered, such as anatomical alignment, kinematic, or functional alignments [6, 7].

Most of these techniques rely primarily on recreating the patient’s lower limb anatomy in the coronal plane, within boundaries with varying restrictiveness, for what has been simplified for years as varus, neutral, and valgus deformities. These are typically defined with the mechanical Hip-Knee-Ankle (mHKA) angle, giving the overall limb alignment. Recently, the Coronal Plane Alignment of the Knee (CPAK) classification has been proposed to broaden alignment understanding and estimate the pre-arthritic knee joint phenotypes [8]. The CPAK classification relies on measures of the long-established mechanical Medial Proximal Tibial Angle (MPTA) and mechanical Lateral Distal Femoral Angle (LDFA) [9] to define the arithmetic HKA (aHKA = MPTA − LDFA) [10] and Joint Line Obliquity (JLO = MPTA + LDFA). Three groups are defined for the JLO angles, and three for the aHKA angles, with thresholds of 0 ± 2° and 180 ± 3°, respectively, a combination of which provides nine coronal knee phenotypes [8].

The growing body of literature related to CPAK currently assesses mostly phenotype distribution in various populations [11–13] and the impact of CPAK types on post-surgical outcomes for different alignment techniques [14–19].

However, the classification’s thresholds were based on experts’ judgment and therefore remain arbitrary. These thresholds do not account for the measurement error of the angles. Indeed, the original studies by McDessi et al. [8, 10] only reported correlations between multiple operators or repeated measurements for the mHKA and aHKA.

A recent study compared the reliability of the CPAK classification assessed with bi-plane and long-leg X-rays [20]. Long-leg X-rays showed superior reliability but still with poor to moderate levels, raising concerns about their suitability for clinical use [21]. In addition, this study found that the smallest detectable changes were in the same range as the CPAK thresholds, suggesting that the classification thresholds may not be adapted to the reliability of the imaging method. Indeed, the reliability of angles and CPAK types should be interpreted together. A second study [22] showed moderate agreement in CPAK classification between two operators in patients with knee OA, with 20% of disagreements, equivalent to a Kappa of 0.6 [21]. Low reliability on the CPAK classification and associated angles could be problematic when using philosophies aiming at restoring native alignment. Indeed, measurement errors could mislead surgical planning and consequent alignment target, which could impact negatively the outcome of surgery despite high surgical accuracy. This highlights the need for a more thorough evaluation of reliability, since the test–retest reliability, the influence of operator experience, or the effect of modifying the thresholds of the CPAK classification were not assessed [20].

Thus, the primary goal of this study was to evaluate the inter-operator, intra-operator, and test–retest reliability of LDFA, MPTA, JLO, aHKA, and CPAK classification in both knees with OA (KOA) and TKA. The secondary goals included assessing the impact of operator experience and the effect of modifying CPAK classification thresholds on its reliability.

We hypothesized that: (1) the reliability of angles and CPAK for KOA will not be suitable for clinical use (ICC < 0.75, SDC > 3°, SEM > 1.1°, Kappa < 0.5) while the reliability for TKA will be (ICC > 0.75, SDC < 3°, SEM < 1.1°; Kappa > 0.6) [21], (2) that the reliability increases with experience, and (3) that increasing CPAK thresholds will improve the classification’s reliability.

## Methods

### Design of experiment

According to the Consensus-based Standards for the selection of health Measurement Instruments (COSMIN) taxonomy [23], the reliability domain of measurement is assessed with repeated measures (inter-rater, intra-rater) and visits (test–retest).

**Fig. 1:**
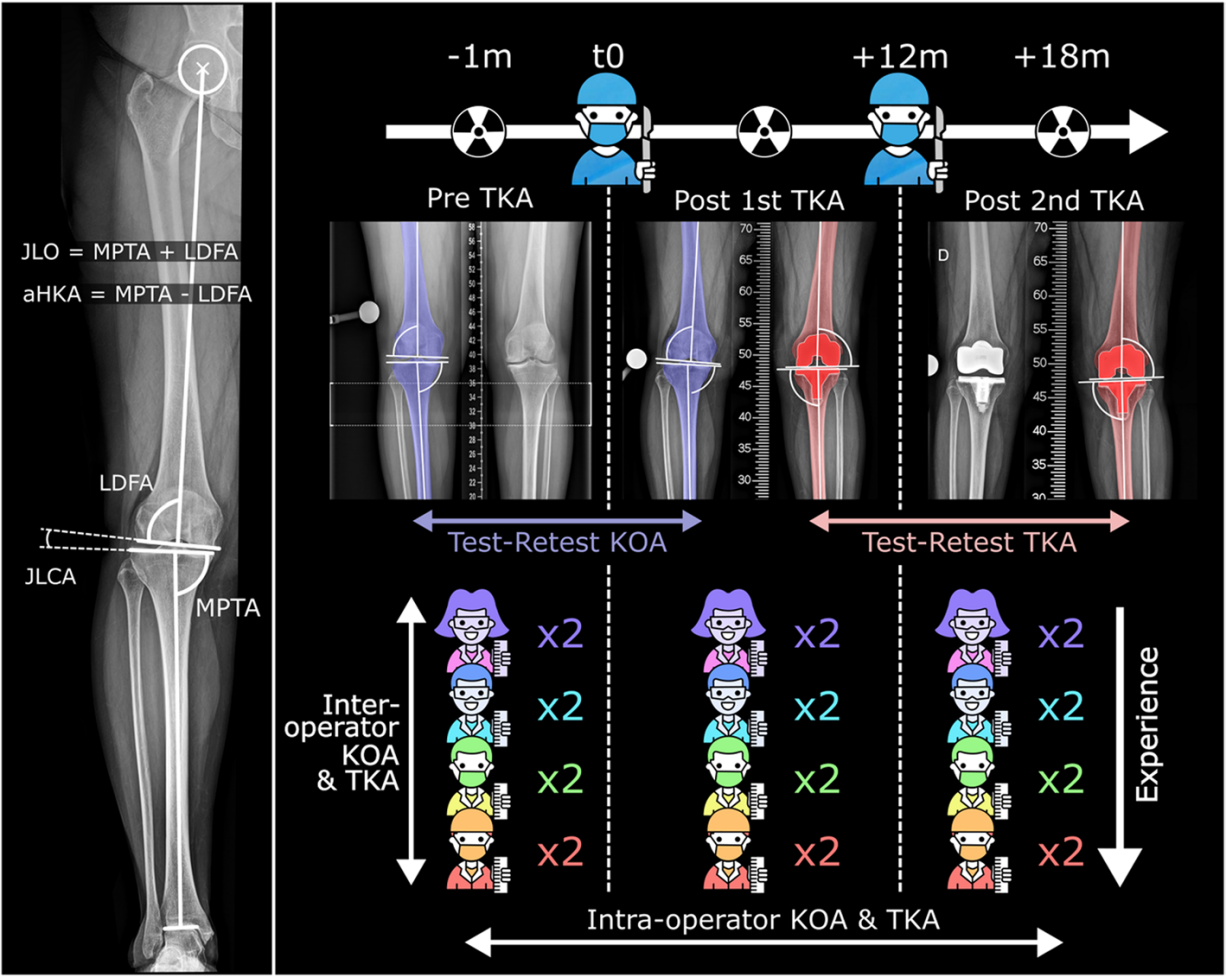
Measured angles and design of experiment. *JLO stands for Joint Line Obliquity, aHKA for arithmetic Hip Knee Ankle angle, MPTA for Medial Proximal Tibial Angle, LDFA for Lateral Distal Femoral Angle, JLCA for Joint Line Crossing Angle, KOA for Knee OsteoArthritis, and TKA for Total Knee Arthroplasty*

The reliability of both osteoarthritic (KOA) and implanted (TKA) knees was assessed on the same population to avoid inter-cohort variability. Patients who underwent consecutive TKA of both knees within 12 to 18 months, between 2018 and 2023, with long-leg X-rays performed before the first TKA (PRE), between TKAs (POST1), and after the second TKA (POST2) were identified from our local registry (Fig. 1).

Due to the short period between X-rays, we assumed that the bony anatomy of the contralateral knee remained unchanged between PRE and POST1 and that the first TKA did not migrate between POST1 and POST2, thus providing identical alignments in both images. Accordingly, the PRE and POST1 X-rays were considered as test–retest for knees with OA when evaluating the contralateral knee, and the POST1 and POST2 X-rays as test–retest for knees with TKA when evaluating the first TKA. Nevertheless, we acknowledge that this study is performing test–retest under real-world imaging variability and that long leg X-rays may not detect subtle OA progression or subtle components migration. The validity of this hypothesis was tested by comparing the levels of reliability of test–retest and intra-rater conditions. Comparable levels would support the hypothesis that the differences between measurements are attributable only to measurement errors and/or variations in patient positioning.

To assess the inter- and intra-rater reliability, two repeated measures were manually performed by each operator for each X-ray with the lower limb deformity workflow of the Traumacad software (Brainlab, Munich, Germany). This software provides a template with a sequence of points and axes to place on the long-axis X-rays, leading to automatic calculation of MPTA and LDFA. The repeated measures were performed with a minimum interval of one week, and the measures were randomized for each operator and each round of measurement. To assess the effect of experience level, four operators with increasing levels of experience were involved. Two engineers were trained to assess CPAK on long-leg X-rays, with respectively 68 and over 200 training cases for OP1 and OP2. The two surgeons (OP3 and OP4) were practicing in a centre with > 400 TKA cases per year and with routine use of CPAK for TKA planning for 6 months (junior orthopaedic surgeon, < 6 years of experience), and 3 years (senior surgeon, > 20 years of experience). Four levels of experience were accordingly defined: low, moderate, high, and expert.

This allowed the evaluation of the reliability (intra-operator, inter-operator, test–retest) of knee angles (LDFA, MPTA, JLO, aHKA) and CPAK classification for knees with OA or with TKA, and for operators with low, moderate, high, and expert levels of experience.

This study was approved by the local Institutional Review Board. All patients provided signed informed consent.

### Data analysis

#### Reliability of angles

According to the COSMIN taxonomy [23], the reliability domain is composed of the reliability construct assessed with the Intraclass Correlation Coefficient (ICC), and of the measurement error assessed with the standard error of measurements (SEM) and smallest detectable change ($\mathrm{SDC}=1.96\times \sqrt{2}\times \mathrm{SEM}$). Since, in clinical practice, measures are typically performed once per X-rays but with multiple operators involved along the patient pathway, a single measure, two-way mixed effects model was used to define the multiple variance components [24], i.e., between-patient, between-operator, between-visit, and between-measure variability. The total variance and various variance components were then used to measure the ICCs, SEMs, and SDCs, with a detailed method published previously [24, 25]. The ICCs [26] were classified as excellent (> 0.9), good (0.75 to 0.9), moderate (0.5 to 0.75), and poor (< 0.5) [27] and were of type (3,1). The SDCs were classified as excellent (< 2°), good (2° to 3°), moderate (3° to 5°), and poor (> 5°), and consequently SEM as excellent (< 0.7°), good (0.7° to 1.1°), moderate (1.1° to 1.8°), and poor (> 1.8°). The estimates and 95% Confidence Interval of ICC, SEM, and SDC were computed with the bootstrap technique (2000 iterations) and the percentile method [28]. Finally, statistical differences were assessed between SDCs assessed in KOA and TKA conditions (paired Student’s *t*-test), between SDCs in intra-operator, inter-operator, and test–retest conditions (one-way Anova with post-hoc paired Student’s *t*-test), and finally between operators (one-way Anova with post-hoc paired Student’s *t*-test). Significance level was set at *p* < 0.05.

The effect of experience was assessed by evaluating intra-operator and test–retest reliability for each operator with the same statistical model, only excluding the inter-operator source of variance.

These analyses were performed in R-4.4.2 with the *lme4* and *boot* packages [29–31].

#### Reliability of CPAK classification

According to the COSMIN taxonomy, the reliability of nominal classification should be assessed with Cohen’s Kappa [23]. Intra-rater variability was assessed with Cohen’s Kappa by comparing the first measurement of each operator to their second measurement, with each measurement considered as an individual observation. The inter-rater variability was assessed using Fleiss’s Kappa by comparing the first measure of each of the four operators for each X-ray. Finally, the test–retest reliability was assessed with Cohen’s Kappa by comparing the first measure from the first visit (PRE for KOA, POST1 for TKA) with the first measure of the second visit (POST1 for KOA, POST2 for TKA). Intra-operator and test–retest reliability were assessed for each operator using the same method and compared to evaluate the effect of experience. The agreements measured with Kappa were classified as slight (0 to 0.2), fair (0.2 to 0.4), moderate (0.4 to 0.6), substantial (0.6 to 0.8), and almost perfect (> 0.8) [32]. Kappa values above 0.6 were deemed appropriate for clinical use, inappropriate below 0.5, and a Kappa score in the range 0.5 to 0.6 as potentially good quality that may be improved [21].

The measurements’ reliability was visualized in the CPAK space by plotting 95% confidence ellipses regrouping all measures for one patient and one condition (KOA and TKA).

#### Impact of modifying the range of classification

The effect of changing the range of the classification on its reliability was assessed by computing the intra-rater, inter-rater, and test–retest reliability of classification with three arbitrary increments of + 1° for the thresholds of aHKA (± 3°, ± 4°, ± 5°) and JLO (± 4°, ± 5°, ± 6°).

#### Power analysis

Bouché et al. found minimal Kappa values of 0.31 [20]; to be safe, a minimal Kappa of 0.2 was targeted. With 9 categories and a power of 0.8, 35 patients are required to assess this Kappa level, while 17 patients are required for a Kappa of 0.3 [33].

#### Population characteristics

This study included 34 patients undergoing primary TKA (20 women, mean [standard deviation]: 67.2 [7.3] years old, BMI: 32.0 [5.6] kg/m^2^, Table 1). One knee with OA and one knee with TKA were selected per patient, two images per knee (on long-leg X-rays) were used, and two measures were performed for each image by four operators, leading to a total of 1088 measures included in the study: 544 for the KOA analysis and 544 for the TKA analysis.

**Table 1:** Characteristics of the population

	**Included patients *n* = 34**
**Sex**	
Women	20 (59%)
Men	14 (41%)
**Age at operation**	67.2 (7.3)
**BMI** (kg/m^2^)	32.0 (5.6)
**ASA Scores**	
1	0 (0%)
2	28 (82%)
3	6 (18%)
4	0 (0%)
**Kellgren-Lawrence Scores**	
1	0 (0%)
2	5 (15%)
3	9 (26%)
4	20 (59%)
**Type of Osteoarthritis**	
Primary OA	33 (97%)
Post-meniscectomy	1 (3%)
**Location of OA**	
Medial/Lateral/Patellar	30 (88%)
Lateral/Patellar	1 (3%)
Medial/Patellar	2 (6%)
Medial	1 (3%)

Values are count (%) or mean (standard deviation)

## Results

The average time between X-rays was 10.6 ± 4.6 months for knees with OA and 12.6 ± 3.5 months for knees with TKA.

### Reliability of angles

The SDCs in TKA conditions were good to excellent, while they were moderate to excellent for KOA, with significant differences (*p* < 0.001, Table 2). More specifically, MPTA showed worse results than LDFA for KOA, up to two times higher for inter-rater SDCs. *T*he angles showed good to excellent ICCs apart from JLO for KOA (moderate, Table 3). There were no significant differences between intra-operator, inter-operator, and test–retest reliabilities (*p* = 0.090).

**Table 2:** Standard error of measurement and smallest detectable change for long-axis radiographic angles across intra-operator, inter-operator, and test–retest conditions

	**Descriptive**				**SEM [95% CI]**			**SDC [95% CI]**			
	**Mean**	**SD**	**Min**	**Max**	**Intra**	**Inter**	**Trtest**	**Intra**	**Inter**		**Trtest**
**Osteoarthritic knee**											
LDFA	88.9	2.3	84	95	0.6 [0.5–0.6]	0.7 [0.6–0.8]	0.8 [0.7–0.8]	1.8 [1.4–1.7]	2.0 [1.8–2.1]		2.3 [1.9–2.3]
MPTA	87.4	2.7	78	94	0.9 [0.7–0.8]	1.4 [1.2–1.4]	1.2 [1.1–1.3]	2.4 [1.9–2.3]	3.8 [3.4–3.9]		3.4 [2.9–3.5]
JLO	176.3	2.8	168	186	1.1 [0.8–1.0]	1.5 [1.4–1.6]	1.5 [1.3–1.6]	3.1 [2.3–2.9]	4.2 [3.8–4.4]		4.2 [3.6–4.4]
aHKA	1.4	4.1	− 9	12	1.1 [0.8–1.0]	1.6 [1.4–1.6]	1.4 [1.2–1.5]	3.0 [2.3–2.8]	4.3 [3.9–4.5]		4.0 [3.4–4.0]
**Knee with total knee arthroplasty**											
LDFA	90.0	2.3	83	95	0.5 [0.4–0.5]	0.7 [0.6–0.7]	0.8 [0.6–0.8]	1.4 [1.1–1.4]	1.9 [1.7–2.0]	2.1 [1.8–2.2]	
MPTA	89.6	2.1	84	95	0.5 [0.4–0.5]	0.6 [0.5–0.7]	0.7 [0.6–0.7]	1.4 [1.0–1.5]	1.8 [1.5–1.9]	1.9 [1.6–2.0]	
JLO	179.5	3.0	173	188	0.7 [0.5–0.7]	1.0 [0.9–1.1]	1.0 [0.9–1.1]	2.1 [1.5–2.1]	2.9 [2.5–3.1]	2.9 [2.4–3.0]	
aHKA	0.4	3.2	− 9	9	0.7 [0.6–0.7]	0.8 [0.7–0.9]	1.0 [0.8–1.0]	2.0 [1.5–1.9]	2.3 [2.0–2.4]	2.8 [2.3–2.9]	

Results are presented as estimates [95% confidence interval]. The criteria are defined as follows: for SEM, Excellent (0.7°), Good (0.7° to 1.1°), Moderate (1.1° to 1.8°), and Poor (> 1.8°); for SDC, Excellent (< 2°), Good (2° to 3°), Moderate (3° to 5°), and Poor (> 5°)

*Abbreviations:* SEM, standard error of measurement; SDC, smallest detectable change; Intra, intra-operator; Inter, inter-operator; Trtest, test–retest; LDFA, lateral distal femoral angle; MPTA, medial proximal tibial angle; JLO, joint line obliquity; aHKA, arithmetic hip-knee-ankle angle

**Table 3:** Intraclass correlation coefficients for long-axis radiographic angles across intra-operator, inter-operator, and test–retest conditions

	**ICC**		
	**Intra**	**Inter**	**Trtest**
**Osteoarthritic knee**			
LDFA	0.923 [0.929–0.955]	0.900 [0.891–0.924]	0.874 [0.873–0.912]
MPTA	0.897 [0.908–0.940]	0.753 [0.734–0.794]	0.796 [0.787–0.851]
JLO	0.852 [0.870–0.915]	0.721 [0.695–0.774]	0.715 [0.703–0.794]
aHKA	0.935 [0.941–0.962]	0.859 [0.847–0.885]	0.882 [0.879–0.915]
**Knee with total knee arthroplasty**			
LDFA	0.952 [0.956–0.973]	0.915 [0.903–0.937]	0.898 [0.891–0.926]
MPTA	0.940 [0.937–0.968]	0.906 [0.890–0.933]	0.891 [0.882–0.926]
JLO	0.942 [0.942–0.969]	0.886 [0.870–0.912]	0.884 [0.876–0.918]
aHKA	0.950 [0.954–0.971]	0.933 [0.928–0.950]	0.904 [0.900–0.932]

Results are presented as estimates [95% confidence interval]. The criteria are defined as follows: for ICC, Excellent (> 0.9), Good (0.75 to 0.9), Moderate (0.5 to 0.75), Poor (< 0.5)

*Abbreviations:* ICC, intraclass correlation coefficient; Intra, intra-operator; Inter, inter-operator; Trtest, test–retest; LDFA, lateral distal femoral angle; MPTA, medial proximal tibial angle; JLO, joint line obliquity; aHKA, arithmetic hip-knee-ankle angle

### Effect of experience

Significant differences were found between operators (*p* < 0.001). OP1 had the lowest reliability (*p* < 0.001), OP3 showed the highest (*p* < 0.001), while OP2 and OP4 did not have a significant difference between them (*p* = 0.237, Tables 4 and 5).

**Table 4:** Intraclass correlation coefficients for intra-operator and test–retest conditions stratified by operator level of experience

	**Knee with Osteoarthritis**		**Knee with Total Knee Arthroplasty**	
	**ICC**		**ICC**	
	**Intra**	**Trtest**	**Intra**	**Trtest**
**LDFA: Lateral Distal Femoral Angle**				
OP1	0.830 [0.766–0.922]	0.795 [0.745–0.892]	0.883 [0.838–0.947]	0.755 [0.646–0.884]
OP2	0.955 [0.933–0.977]	0.907 [0.872–0.944]	0.972 [0.949–0.986]	0.919 [0.876–0.957]
OP3	0.968 [0.951–0.983]	0.910 [0.871–0.948]	0.990 [0.849–0.999]	0.927 [0.267–0.959]
OP4	0.937 [0.914–0.969]	0.869 [0.812–0.931]	0.956 [0.935–0.979]	0.918 [0.887–0.953]
**MPTA: Medial Proximal Tibial Angle**				
OP1	0.837 [0.783–0.924]	0.720 [0.625–0.855]	0.884 [0.851–0.946]	0.823 [0.740–0.908]
OP2	0.894 [0.854–0.955]	0.771 [0.681–0.880]	0.928 [0.848–0.983]	0.898 [0.785–0.951]
OP3	0.964 [0.947–0.980]	0.867 [0.818–0.920]	0.983 [0.842–0.995]	0.919 [0.405–0.951]
OP4	0.893 [0.851–0.949]	0.747 [0.672–0.852]	0.953 [0.931–0.980]	0.923 [0.896–0.955]
**JLO: Joint Line Obliquity**				
OP1	0.770 [0.688–0.891]	0.587 [0.472–0.778]	0.865 [0.809–0.940]	0.743 [0.622–0.893]
OP2	0.877 [0.835–0.942]	0.743 [0.646–0.869]	0.944 [0.888–0.982]	0.901 [0.850–0.950]
OP3	0.934 [0.909–0.966]	0.788 [0.724–0.862]	0.986 [0.934–0.994]	0.917 [0.736–0.955]
OP4	0.853 [0.806–0.930]	0.699 [0.600–0.828]	0.954 [0.943–0.977]	0.910 [0.875–0.947]
**aHKA: arithmetic Hip Knee Ankle angle**				
OP1	0.868 [0.828–0.933]	0.831 [0.776–0.916]	0.897 [0.880–0.945]	0.822 [0.741–0.906]
OP2	0.945 [0.927–0.973]	0.877 [0.837–0.926]	0.962 [0.939–0.985]	0.916 [0.878–0.961]
OP3	0.978 [0.969–0.989]	0.921 [0.899–0.948]	0.987 [0.901–0.994]	0.929 [0.698–0.967]
OP4	0.945 [0.928–0.970]	0.863 [0.822–0.924]	0.956 [0.935–0.979]	0.934 [0.910–0.966]

Results are presented as estimates [95% confidence interval]. The criteria are defined as follows: for ICC, Excellent (> 0.9), Good (0.75 to 0.9), Moderate (0.5 to 0.75), Poor (< 0.5)

*Abbreviations:* ICC, intraclass correlation coefficient; Intra, intra-operator; Trtest, test–retest

**Table 5:** Standard error of measurement and smallest detectable change for intra-operator and test–retest conditions stratified by operator level of experience

	**Knee with Osteoarthritis**				**Knee with Total Knee Arthroplasty**			
	**SEM**		**SDC**		**SEM**		**SEM**	
	**Intra**	**Trtest**	**Intra**	**Trtest**	**Intra**	**Trtest**	**Intra**	**Trtest**
**LDFA: Lateral Distal Femoral Angle**								
OP1	0.9 [0.6–1.1]	1.0 [0.7–1.1]	2.5 [1.7–2.9]	2.7 [2.0–3.0]	0.7 [0.4–0.8]	0.9 [0.7–1.2]	1.8 [1.2–2.1]	2.6 [1.8–3.2]
OP2	0.5 [0.4–0.6]	0.7 [0.6–0.9]	1.4 [1.0–1.7]	2.0 [1.6–2.4]	0.4 [0.3–0.5]	0.7 [0.5–0.9]	1.2 [0.8–1.4]	2.0 [1.4–2.4]
OP3	0.4 [0.3–0.5]	0.7 [0.5–0.8]	1.2 [0.8–1.4]	2.0 [1.5–2.3]	0.2 [0.1–0.6]	0.6 [0.4–1.3]	0.7 [0.3–1.6]	1.8 [1.1–3.6]
OP4	0.6 [0.4–0.7]	0.8 [0.6–1.0]	1.6 [1.1–1.8]	2.3 [1.6–2.7]	0.5 [0.4–0.6]	0.7 [0.6–0.8]	1.5 [1.0–1.8]	2.0 [1.5–2.3]
**MPTA: Medial Proximal Tibial Angle**								
OP1	1.1 [0.8–1.3]	1.4 [1.0–1.7]	3.0 [2.1–3.5]	4.0 [2.9–4.6]	0.7 [0.5–0.7]	0.8 [0.6–1.0]	1.8 [1.2–2.1]	2.2 [1.6–2.7]
OP2	0.9 [0.6–1.0]	1.3 [0.9–1.5]	2.4 [1.6–2.9]	3.6 [2.6–4.2]	0.6 [0.3–0.9]	0.7 [0.5–1.0]	1.6 [0.8–2.4]	2.0 [1.3–2.7]
OP3	0.6 [0.4–0.7]	1.1 [0.8–1.2]	1.6 [1.1–1.9]	3.0 [2.3–3.4]	0.3 [0.1–0.5]	0.6 [0.4–1.0]	0.8 [0.4–1.4]	1.6 [1.1–2.7]
OP4	0.7 [0.5–0.9]	1.1 [0.9–1.3]	2.0 [1.4–2.4]	3.1 [2.4–3.5]	0.5 [0.3–0.6]	0.6 [0.5–0.7]	1.3 [0.8–1.6]	1.7 [1.3–1.9]
**JLO: Joint Line Obliquity**								
OP1	1.4 [1.0–1.6]	1.9 [1.4–2.1]	3.8 [2.7–4.4]	5.1 [3.8–5.9]	0.9 [0.6–1.1]	1.3 [0.8–1.6]	2.5 [1.7–3.0]	3.5 [2.3–4.4]
OP2	1.1 [0.7–1.2]	1.5 [1.1–1.8]	2.9 [2.0–3.4]	4.3 [3.0–5.0]	0.8 [0.4–1.1]	1.0 [0.7–1.3]	2.2 [1.2–3.0]	2.9 [2.0–3.5]
OP3	0.7 [0.5–0.8]	1.3 [1.1–1.5]	2.0 [1.5–2.3]	3.6 [3.0–4.1]	0.4 [0.2–0.5]	0.9 [0.6–1.1]	1.0 [0.6–1.4]	2.5 [1.8–3.1]
OP4	1.0 [0.7–1.1]	1.4 [1.1–1.6]	2.7 [1.9–3.1]	3.9 [2.9–4.5]	0.7 [0.5–0.8]	1.0 [0.7–1.1]	1.9 [1.3–2.1]	2.7 [2.1–3.1]
**aHKA: arithmetic Hip Knee Ankle angle**								
OP1	1.4 [1.0–1.7]	1.6 [1.2–1.9]	4.0 [2.9–4.6]	4.5 [3.2–5.3]	0.9 [0.7–1.0]	1.2 [0.9–1.5]	2.6 [1.9–2.8]	3.4 [2.5–4.2]
OP2	1.0 [0.7–1.1]	1.4 [1.1–1.7]	2.7 [1.9–3.1]	4.0 [3.1–4.6]	0.7 [0.4–0.8]	1.0 [0.7–1.2]	1.8 [1.2–2.3]	2.7 [1.9–3.3]
OP3	0.7 [0.5–0.8]	1.3 [1.1–1.5]	1.9 [1.4–2.2]	3.6 [3.0–4.0]	0.4 [0.2–0.6]	0.8 [0.5–1.1]	1.0 [0.6–1.7]	2.4 [1.5–3.0]
OP4	0.9 [0.6–1.0]	1.4 [1.0–1.5]	2.4 [1.8–2.7]	3.8 [2.9–4.3]	0.7 [0.5–0.9]	0.9 [0.6–1.0]	2.0 [1.4–2.4]	2.5 [1.8–2.8]

Results are presented as estimates [95% confidence interval]. The criteria are defined as follows: for SEM, Excellent (0.7°), Good (0.7° to 1.1°), Moderate (1.1° to 1.8°), and Poor (> 1.8°); for SDC, Excellent (< 2°), Good (2° to 3°), Moderate (3° to 5°), and Poor (> 5°)

*Abbreviations:* SEM, standard error of measurement; SDC, smallest detectable change; Intra, intra-operator; Trtest, test–retest

### Reliability of the CPAK classification

**Fig. 2:**
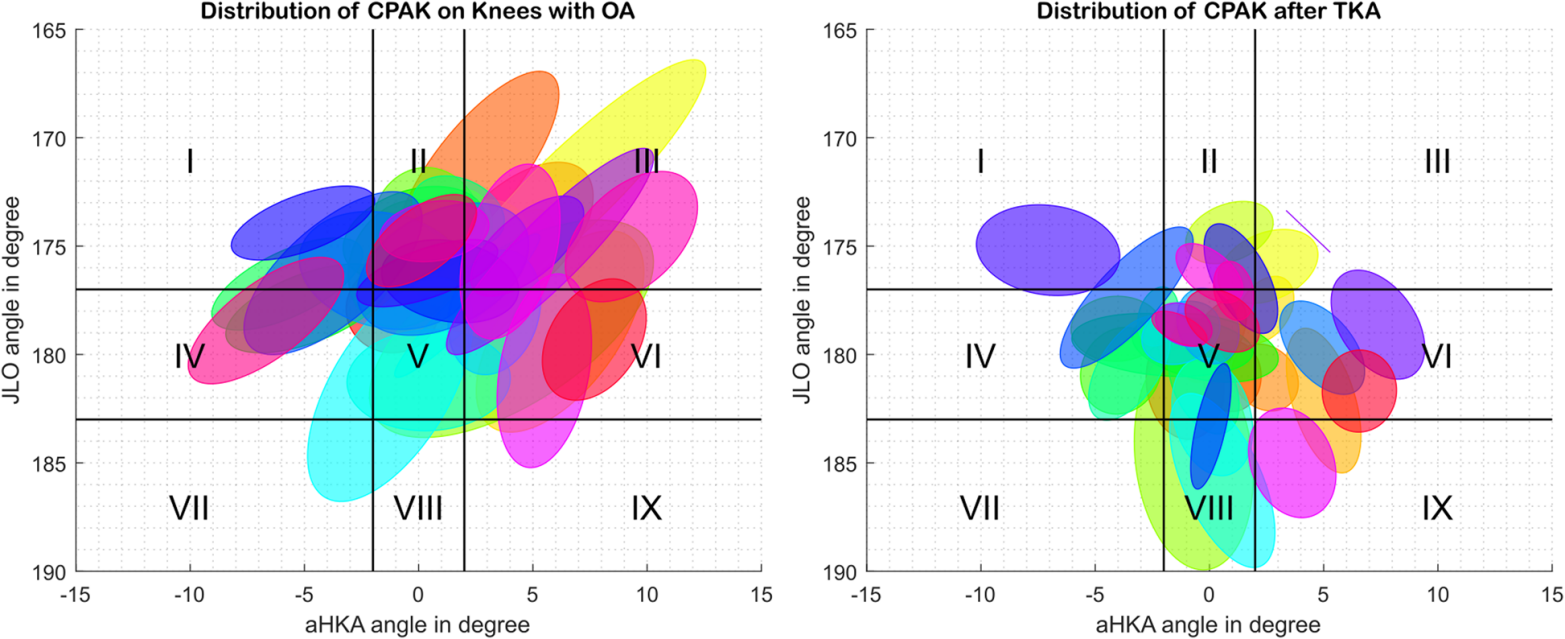
Representation of all patients on the CPAK space. *Each ellipse represents the 95%CI over the measures of all operators. KOA stands for knee with osteoarthritis, and TKA stands for knee with total knee arthroplasty*

The variability of knee alignment measures was pictured in Fig. 2 for KOA (Fig. 2a) and TKA conditions (Fig. 2b).

The classification as defined by McDessi et al. [8] showed substantial intra-operator reliability and moderate inter-operator and test–retest reliability in KOA conditions (Table 6), while it was substantial to almost perfect in TKA conditions. Test–retest reliability was similar to inter-operator reliability but lower than intra-operator reliability.

**Table 6:** Reliability of the standard CPAK classification, including the influence of operator experience and the effect of increasing classification margins

		**Intra**	**Inter**	**Trtest**
**Standard Classification**				
KOA		0.64 [0.57–0.70]	0.55 [0.46–0.65]	0.50 [0.39–0.59]
TKA		0.81 [0.76–0.87]	0.74 [0.66–0.82]	0.69 [0.59–0.78]
**Effect of Experience**				
**KOA**	OP1	0.52 [0.38–0.67]	-	0.46 [0.23–0.68]
	OP2	0.57 [0.43–0.71]	-	0.36 [0.17–0.55]
	OP3	0.81 [0.70–0.91]	-	0.55 [0.34–0.74]
	OP4	0.62 [0.49–0.75]	-	0.58 [0.39–0.77]
**TKA**	OP1	0.61 [0.45–0.76]	-	0.59 [0.35–0.81]
	OP2	0.86 [0.76–0.95]	-	0.73 [0.55–0.89]
	OP3	0.86 [0.74–0.95]	-	0.71 [0.51–0.88]
	OP4	0.88 [0.78–0.96]	-	0.68 [0.48–0.87]
**Effect of Increasing margins**				
**KOA**	CPAK + 1	0.64 [0.57–0.70]	0.50 [0.40–0.61]	0.43 [0.32–0.54]
	CPAK + 2	0.59 [0.51–0.67]	0.44 [0.33–0.54]	0.50 [0.38–0.62]
	CPAK + 3	0.67 [0.58–0.75]	0.49 [0.37–0.61]	0.61 [0.48–0.73]
**TKA**	CPAK + 1	0.79 [0.72–0.86]	0.78 [0.66–0.87]	0.64 [0.52–0.75]
	CPAK + 2	0.77 [0.69–0.85]	0.70 [0.58–0.82]	0.52 [0.37–0.66]
	CPAK + 3	0.79 [0.67–0.90]	0.67 [0.49–0.82]	0.59 [0.36–0.78]

Results are presented as estimates [95% confidence interval]. The criteria are defined as follows: Almost Perfect (> 0.8), Substantial (0.6 to 0.8), Moderate (0.4 to 0.6), Fair (0.2 to 0.4), Slight (< 0.2)

OP3 showed higher reliability on KOA, while OP1 showed the lowest reliability for TKA, with other operators at the same level in both cases (Table 6).

Changing the thresholds of the classification did not improve reliability.

## Discussion

This study evaluated the reliability of the CPAK classification and associated angles, as well as the impact of modifying CPAK type ranges for knees with OA and knees with TKA. Inter-operator, intra-operator, and test–retest reliabilities were assessed, along with the effect of experience by considering four levels of experience (low, moderate, high, and expert level).

The first hypothesis was rejected. Indeed, the reliability of the CPAK classification was moderate for KOA and substantial to almost perfect for TKA knees, underlining its potential use in clinical practice, particularly in the postoperative setting. Indeed, for KOA, the Kappas between 0.5 and 0.6 imply a reliability of “potentially good quality” with a range for potential improvement [21].

In accordance with the second hypothesis, experience improved CPAK reliabilities. Indeed, higher reliability was found among surgeons compared to trained engineers. Still, surgeons’ test–retest reliabilities remained moderate and approached the threshold for clinical acceptability [21], suggesting room for improvement.

Contrary to our third hypothesis, modifying the original thresholds [8] did not improve the reliability of the CPAK classification, potentially linked to well-known limitations of categorising continuous variables [34]. Indeed, with repeated measures, patients close to the threshold will always fall between multiple categories, as pictured in Fig. 2. Modifying thresholds will only change the patients falling in between categories but will not improve the intrinsic reliability of the classification. Thus, regarding these results and the MDCs of angles, classifying patients in CPAK types with the thresholds defined for aHKA and JLO by Mc Dessi et al. [8] seems reasonable. An alternative approach could be to provide a likelihood score for each patient based on SDCs. For example, a patient might have an 80% chance of being classified as type I and a 20% chance of being classified as type II. This may help illustrate that the anatomy of a Type I patient with a JLO of 178° and an aHKA of −3° may be closer to that of a Type V patient than to another Type I patient at the opposite end of the spectrum, with a JLO of 168° and an aHKA of −10°. This range in CPAK types may limit the use of CPAK classification in everyday practice, as underlined in two other studies that assessed intra- and inter-operator reliability in a population mixing patients with knee OA and TKA [20] and the inter-operator reliability in knees with OA while excluding patients with bilateral OA [22]. Planning based on the CPAK type only may lead to different surgical decisions depending on the operator. However, regarding the SDCs and MDCs presented and the good reproducibility of angles in the aforementioned studies [20, 22], the direct use of angular measurements may be more appropriate for reconstructing and understanding patients’ native phenotypes more accurately. Still, the impact of the SDC on the surgical planning and decision was beyond the scope of the present study and may be evaluated in the future.

**Fig. 3:**
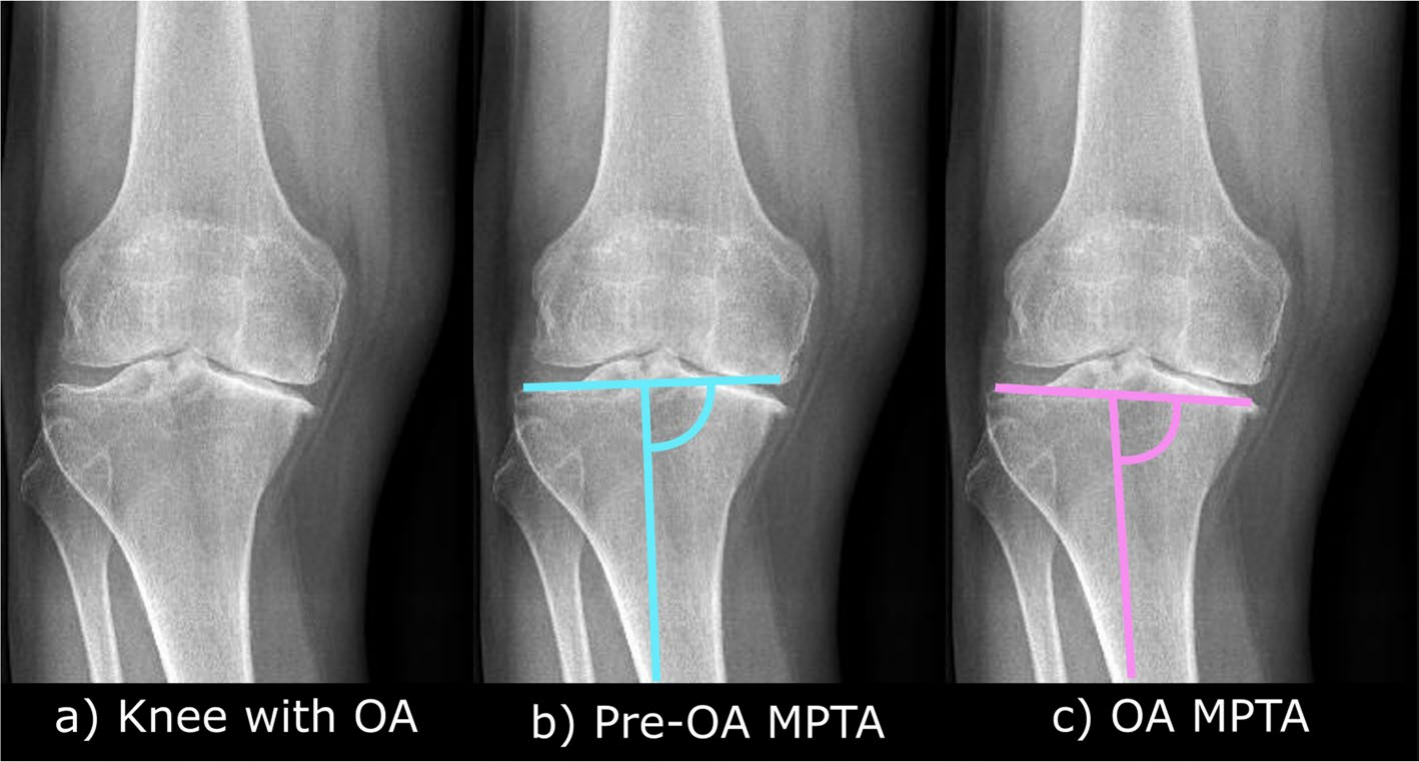
Range of MPTA choice for OA knees

Regarding the angles, LDFA showed similar reliability between KOA and TKA conditions, whereas MPTA in KOA showed approximately 2 times higher SDCs compared to the LDFA in KOA and the MPTA in TKA. Thus, MPTA in knees with OA appeared as the main factor undermining reliability, potentially due to different interpretations caused by bone loss. For example, Fig. 3 shows a knee with OA and bone loss, where assessing MPTA might be challenging: one approach may focus on identifying the pre-arthritic tibia anatomy (blue line, Fig. 3b), while another may prioritize measuring the arthritic bony anatomy (purple line, Fig. 3c). Interestingly, after discussion between operators, it was noted that, despite defining common rules before measurements, OP4 tended to focus on the pre-arthritic MPTA (Fig. 3b) while OP3 tended to go toward OA MPTA (Fig. 3c) with slightly higher reliability. However, assessing differences in angle between the pre-arthritic and OA MPTA, as well as their impact on surgery planning and outcome, would require a different study design.

The main limitation of the present study was the high percentage of patients with a Kellgren-Lawrence level of 4 (59%). Many presented with concomitant bone wear, complicating the definition of the pre-arthritic MPTA and likely reducing the reliability of our measurements. Targeting patients with bilateral knee OA may have induced a selection bias toward patients with severe OA. However, this sample from our local registry database was representative of daily clinical practice when assessing patients planned for TKA. It would be expected that knees with lower OA and limited bone wear show higher reliability levels that would be acceptable for clinical use. However, an evaluation of reliability stratified by OA severity would be required to clarify this issue, but it was beyond the scope of the present study. The original CPAK definition did underline that using the aHKA to determine the pre-arthritic alignment was only possible “In the absence of arthritic bone loss at the central compartmental contact points” [8, 10]. Therefore, the severity of OA, specifically the presence of bone wear, seems to be an intrinsic limitation of the CPAK classification. Nevertheless, the present reliability levels showed the feasibility of assessing the pre-arthritic MPTA consistently with sufficient training, even in those cases. A clarification of the MPTA definition in knees with severe OA may help improve the reliability and target the correct pre-arthritic alignment.

Another limitation was using long-leg X-rays acquired at different time points for the test–retest assessment. Despite the close time interval between X-rays (overall average of 11.6 ± 4 months), the severity of OA might have changed. Nevertheless, by definition, the classification and angles should only be affected by bone loss [8, 10]. Therefore, progression in joint space narrowing was not considered a limitation, while significant bone loss progression was unlikely in such a time period. Indeed, accelerated knee osteoarthritis appears to be a unique subset of knee OA with the presence of injury, greater age, higher BMI, and knee pain as potentially associated risk factors [35]. More precisely, age and BMI seem to play a role in patients younger than 63.5 years old, whereas glucose levels and static alignment seem to play a role in patients older than 63.5 years old for the development of knee OA within 4 years [35]. Glucose levels for our cohort were not available, but the mean age of 67.2 years old and the mean varus alignment (> 2.3°) made them at risk for accelerated knee OA. Regarding the test–retest assumption for knees with TKA, the present series contained only patients with cemented TKA, making implant migration unlikely; flexion contractures might have impacted the appreciation of the angles. Moreover, there were no differences in reliability levels between intra-operator, inter-operator, and test–retest conditions, which appear to validate our assumption. Indeed, this means that differences in angles between test–retest X-rays were of the same magnitude as differences between operators for the same image. Thus, strong OA progression or implant migration was not present in our series. This also suggests proper standardisation of long leg X-rays between measurements and consistency across visits, allowing for the possibility of following patients over time and comparing pre- and post-surgery changes. This is consistent with current practice in our centre, where patients’ positioning when acquiring long-leg X-Rays is routinely checked for reproducibility (at ease position, patella centered).

The pre-surgery distribution of CPAK type in our population was mostly toward type III and valgus knees, while most studies present a majority of patients in type I and II [12] and a shift toward type I with increasing Kellgren-Lawrence scores [36]. This difference may reflect a selection bias toward patients with advanced bilateral knee OA, resulting from the study design constraint of avoiding additional radiation for the patients while assessing test–retest reliability. The CPAK phenotypes assessed in this study might not represent the distribution of the whole cohort.

Finally, the power analysis recommended 35 patients to be able to assess a minimal Kappa of 0.2 for a scale with 9 categories and a power of 0.8, but only 34 patients were included. Regarding the fact that the minimal Kappa estimate was 0.36, that 39 of 40 Kappas computed for this study were above 0.4, and that 17 patients only are required to assess a Kappa of 0.3 with 9 categories and a power of 0.8 [33], we assumed the statistical power was sufficient with 34 patients.

## Conclusions

For experienced operators, the CPAK classification and associated angles demonstrated reliability levels appropriate for clinical use in knees with TKA, while knees with severe OA approached the threshold of acceptability. In severe OA, CPAK classification should be interpreted cautiously, and angular measures may be preferable. MPTA in knees with OA emerged as the principal factor undermining reliability, suggesting that clarification of MPTA measurement in the presence of bony wear should improve reliability and clinical applicability. This is particularly relevant when using the CPAK classification and its angles with philosophies aiming at restoring the native alignment. Test–retest reliability levels suggested that these measures are appropriate for longitudinal assessment.

## Data Availability

The datasets analysed during the current study are publicly available on Yareta with the following 10.26037/yareta:zd6lopqfincqbborhfh2jhb2mu

## Abbreviations

BMI: Body Mass Index
COSMIN: Consensus-based Standards for the selection of health Measurements INstruments
CPAK: Coronal Plane Alignment of the Knee
aHKA: Arithmetic Hip Knee Ankle angle
mHKA: Mechanical Hip Knee Ankle angle
ICC: Intraclass Correlation Coefficient
JLO: Joint Line Obliquity
KOA: Knee with OsteoArthritis
LDFA: Lateral Distal Femoral Angle
MPTA: Medial Proximal Tibial Angle
OA: OsteoArthritis
OP1 to 4: Operator 1 to 4
PRE: Time point before the 1st TKA
POST1: Time point between the 1st and 2nd TKA
POST2: Time point after the 2nd TKA
SDC: Smallest Detectable Change
SEM: Standard Error of Measurement
TKA: Total Knee Arthroplasty
